# Retrospective study and immunohistochemical analysis of canine mammary sarcomas

**DOI:** 10.1186/1746-6148-9-248

**Published:** 2013-12-09

**Authors:** Izabella Dolka, Rafał Sapierzyński, Magdalena Król

**Affiliations:** 1 Department of Pathology and Veterinary Diagnostics, Faculty of Veterinary Medicine, Warsaw University of Life Sciences-WULS, Nowoursynowska 159c, Warsaw 02-776, Poland; 2 Department of Physiological Sciences, Faculty of Veterinary Medicine, Warsaw University of Life Sciences-WULS, Nowoursynowska 159c, Warsaw 02-776, Poland

**Keywords:** Canine mammary tumor, Sarcomas, Immunohistochemistry, Dogs

## Abstract

**Background:**

Canine mammary sarcomas (CMSs) are rarely diagnosed in female dogs, which explains the scarcity of immunohistochemical findings concerning those tumors. This paper presents the results of a retrospective study into CMSs and discusses the clinical features of the analyzed tumors, the expression of intermediate filaments CK, Vim, Des and α-SMA, and the expression of p63, Ki67, ERα, PR and p53 protein.

**Results:**

Four percent of all canine mammary tumors (CMTs) were classified as CMSs, and they represented 5.1% of malignant CMTs. The mean age at diagnosis was 11.1 ± 2.8 years. Large breed dogs were more frequently affected (38.7%). The majority of observed CMSs were fibrosarcomas (2.1%). All CMSs expressed vimentin, and higher levels of vimentin expression were noted in fibrosarcomas and osteosarcomas. Ki67 expression was significantly correlated with the grade of CMS.

**Conclusions:**

Our results revealed that CMSs form a heterogeneous group, therefore, immunohistochemical examinations could support differential and final diagnosis. Although this study analyzed a limited number of samples, the reported results can expand our knowledge about CMSs. Further work is required in this field.

## Background

Canine mammary tumors (CMTs) are the most common neoplasms that account for nearly one-half of all tumors diagnosed in female dogs. Approximately 41% to 53% of CMTs are malignant [1,2]. Most CMTs are epithelial in origin, and they have been extensively researched [3,4]. However, little is known about canine mammary sarcomas (CMSs), which are malignant tumors originating in mesenchymal tissue of the mammary gland, including osteosarcoma, chondrosarcoma, fibrosarcoma and hemangiosarcoma [5,6]. They are considered to have a very poor prognosis and, therefore, pose a great challenge in veterinary practice [1,6,7]. Mammary sarcomas are more often diagnosed in dogs than humans where breast sarcomas constitute less than 1% of malignant breast tumors [8]. The discussed tumors are very rare, they remain poorly investigated, and the majority of published studies into CMSs involve case reports or experiments performed on a small number of samples [7,9-12].

To the best of our knowledge, this study is the first ever immunohistochemical analysis of CMSs. The aim of this research was two-fold: to perform a retrospective analysis (1996–2012) into the percentage of CMSs in all CMTs, to determine the age and breed of affected females, and to evaluate the expression of intermediate filaments: vimentin (Vim), cytokeratin (CK), alpha smooth muscle actin (α-SMA) and desmin (Des) as well as the expression of p63 protein, Ki67 antigen, estrogen receptor alpha (ERα), progesterone receptor (PR), and p53 protein by immunohistochemical staining.

## Methods

### Tumor samples and histopathological examination

In this study, all cases of CMTs described in the archives (1996–2012) of the Division of Animal Pathomorphology at the Department of Pathology and Veterinary Diagnostics have been analyzed. The histological type of the tumor was assessed based on the World Health Organization (WHO) Histological Classification of the Mammary Tumors of the Dog and Cat, the histological grade was based on the assessment of tubule formation, the degree of differentiation and the mitotic index [13,14]. For the needs of this study, CMTs were classified as benign or malignant, and were further subdivided into carcinomas and sarcomas. The age at diagnosis, the affected animal breed and the morphological features of the tumor, including size, location and histological type, were recorded. Eighteen paraffin-embedded specimens of canine mammary sarcomas were analyzed*.* The final diagnosis was based on IHC results and tumor grade. The histological type of CMS was determined based on the proposed criteria [6] and the World Health Organization classification [14]. The mitotic index (MI) was determined as the mean number of cells in mitosis evaluated in 10 high-power fields (HPF) under 40x objective lens (field area 0.239 mm^2^) [15]. A specific grading system for CMSs has not yet been established, therefore, the method used in this study was based on the degree of cell differentiation (pleomorphism), the mitotic index and the presence of necrosis. CMSs were classified into two groups: low*-*grade malignancy (well-differentiated and moderately differentiated, I and II) and high*-*grade malignancy (poorly differentiated, III) [16]. Where an agreement on a diagnosis could not be achieved, a round-table discussion was staged using a multi-headed microscope.

### Immunohistochemical examination (IHC)

Eighteen CMS specimens were subjected to immunohistochemical (IHC) analysis. Five micrometer (μm) thick sections were mounted onto a glass slide covered with 2% Silan solution in acetone. After dewaxing in xylene and rehydration in ethanol for antigen retrieval, the slides were heated in a microwave oven in 0.02 M citrate buffer, pH 6.0. After cooling, the sections were incubated in 3% perhydrol solution for 15 minutes to block the endogenous peroxidase reaction. Non-specific binding was blocked by incubation in 5% bovine serum albumin (Sigma Aldrich, Germany). After 30 min, the following primary antibodies (diluted in 1% bovine serum) were used: monoclonal mouse anti-human vimentin, Vim (clone Vim 3B4, Dako, Denmark) diluted 1:50 [17,18], monoclonal mouse anti-human cytokeratin, CK (clone MNF116, Dako, Denmark) diluted 1:50 [17,18], monoclonal mouse anti-human alpha smooth muscle actin, α-SMA (clone 1A4, Dako, Denmark) diluted 1:100 [19], monoclonal mouse anti-human desmin, Des (clone D33, Dako, Denmark) diluted 1:50 [17-19], monoclonal mouse anti-p63 protein (clone 4A4, Santa Cruz Biotechnology, USA) [20], mouse monoclonal anti-human Ki67 (clone MIB-1, Dako, Denmark) diluted 1:75 [15,21,22], rabbit polyclonal anti-human estrogen receptor alpha, ERα (H-184, Santa Cruz Biotechnology, USA) diluted 1:100 [23], ready-to-use mouse monoclonal anti-human progesterone receptor, PR (clone PR10A9, Immunotech, France) [24,25] and rabbit polyclonal anti-human p53 protein (clone FL-393, Santa Cruz Biotechnology, USA) diluted 1:50 [25,26]. The slides were incubated in a humidified chamber for 1 h at room temperature. A biotinylated secondary antibody (EnVision + System-HRP, Dako, Denmark) was used in accordance with the manufacturer’s instructions. The sections were washed, covered with diaminobenzidine chromogen (DAKO) and counterstained with Mayer's hematoxylin for 10 min. They were dehydrated in a graded series of alcohols, cleared in xylene and mounted using the DPX medium (Gurr®, Sigma-Aldrich) and coverslips. Paraffin-embedded neoplastic canine tissues and healthy tissues with known positive reactivity were used as extrinsic and/or intrinsic positive controls. Negative controls were also performed by omitting the primary antibody.

### Scoring of immunohistochemical data

Immunohistochemical analyses involved at least 10 images of sarcomatous areas acquired at x40 HPF (Olympus microscope BX41). Positive immunostaining for Vim, CK, Des and α-SMA was observed as a brown cytoplasmic precipitate, and for p63 – as a brown nuclear precipitate. The number of immunoreactive cells was classified as: **-** = none, ± = slight (positive cells constituted less than 10%); + = moderate (10–50% of cells were positive); and ++ = intense (more than 50% of cells were positive) [20]. The colorimetric intensity of IHC-stained antigen spots was determined in a computer-assisted image analyzer (Olympus Microimage Image Analysis version 4.0 for Windows, USA), and antigen spot color intensity was expressed as mean pixel optical density on a 1–256 scale.

Positive immunostaining for Ki67, ERα, PR and p53 was defined as nuclear pattern (brown precipitate). Antigen density was determined by counting at least 1000 cells in 10 HPF. The number of positive cells was expressed as the percentage of positively stained cells in the total number of cells [21,27]. Areas with necrosis were omitted.

### Statistical analysis

Data was processed in Prism 5.00 software (GraphPad Software, California, USA) using one-way ANOVA, Tukey's HSD (Honestly Significant Difference) post-hoc test, Spearman's and Pearson's correlation. P-values <0.05 (*) were regarded as significant, whereas p-values <0.01 and <0.001 were considered to be highly significant.

## Results

### Retrospective data for CMSs

A total of 841 CMT cases were found in the archives (1996–2012) of the Division of Animal Pathomorphology. CMSs constituted only 4% (34/841) of all CMTs and 5.1% (34/666) of malignant CMTs. The ratio of sarcomas to carcinomas was 1:18 (34/616). Malignant mixed mammary tumors (carcinosarcomas) were excluded from these groups. The mean age at diagnosis in female dogs was 11.1 ± 2.8 years, within a range of 5 to 17 years. The mean tumor size, defined as the largest diameter, was 8.8 ± 6.1 cm (range of 2–20 cm). The fourth left mammary gland was most commonly affected. In the group of purebred dogs, the percentage of CMSs was higher in large breeds (38.7%, n = 12), i.e. German Shepherds (n = 4) and Rottweilers (n = 2), and single CMS cases were noted in the following breeds: St. Bernard, Tosa Inu, Doberman Pinscher, Flat Coated Retriever, Labrador Retriever and German Shorthaired Pointer.

A lower percentage of CMSs was noted in mongrels and small breeds (both 25.8% and n = 8), but it was very rarely encountered in medium-sized dog breeds (9.7%, n = 3). The breed of three dogs was not identified. The histological types of CMSs and their percentage distribution were follows: 2.1% fibrosarcoma (n = 14), 1.1% osteosarcoma (n = 7), 0.9% liposarcoma (n = 6), 0.6% chondrosarcoma (n = 4), 0.3% hemangiosarcoma (n = 2) and 0.2% unclassified sarcoma (n = 1).

### Immunohistochemical analysis of CMSs

A total of 52.9% (n = 18) of CMS specimens, including 8 osteosarcomas, 6 fibrosarcomas, 2 liposarcomas, 1 chondrosarcoma and 1 unclassified sarcoma, were subjected to immunohistochemical examination. Individual cases were placed in a single group termed ‘other sarcomas’.

The analyzed specimens included 16 high-grade and 2 low-grade CMSs. All osteosarcomas, liposarcomas and other sarcomas were high-grade tumors; 66.7% (n = 4) of fibrosarcomas were high-grade, whereas the remaining fibrosarcomas were classified as low-grade. No significant differences were observed between patients' age or breed, tumor size, sarcoma type or degree of malignancy.

**Figure 1:**
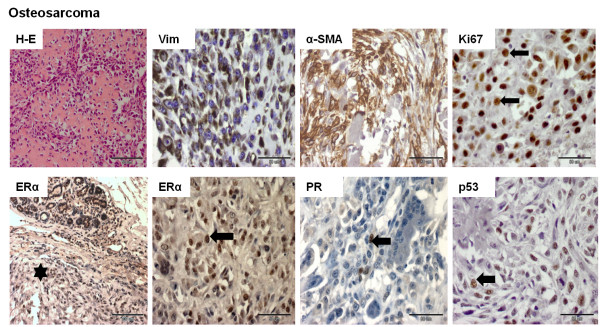
**Histological and immunohistochemical images of canine mammary osteosarcoma.** The histological sample was stained with the standard hematoxylin and eosin (H-E) method. Immunopositivity (nuclear or cytoplasmic) is shown as brown precipitate in neoplastic cells. The EnVision + System-HRP detection system was used, and the signal was visualized with chromogen 3,3-diaminobenzidine 3-3' (DAB). Arrows indicate positive nuclear staining of cells. An asterisk (*) indicates a negatively stained sarcomatous area. Images were obtained under the Olympus BX41 microscope. Original magnification: H-E (x400), Vim (x400), α-SMA (x400), Ki67 (x400), ERα (x200, x400), PR (x400), p53 (x400).

**Figure 2:**
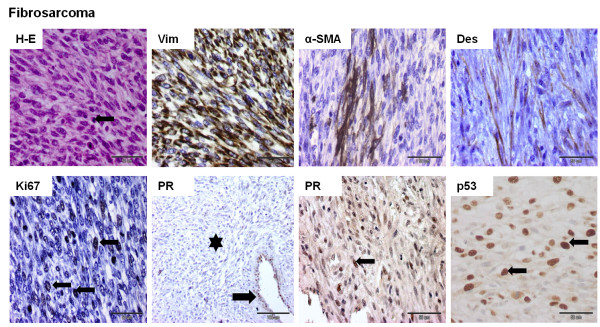
**Histological and immunohistochemical images of canine mammary fibrosarcoma.** The histological sample was stained with the standard hematoxylin and eosin (H-E) method. Positive immunostaining (nuclear or cytoplasmic) was observed as brown precipitate. The EnVision + System-HRP detection system was used, and the signal was visualized with chromogen 3,3-diaminobenzidine 3-3' (DAB). Arrows indicate positive nuclear staining of cells. An asterisk (*) indicates a negatively stained sarcomatous area. Images were obtained under the Olympus BX41 microscope. Original magnification: H-E (x400, arrow indicates a mitotic figure), Vim (x400), α-SMA (x400), Des (x400), Ki67 (x400), PR (x200; arrow indicates PR-positive epithelial cells of the mammary gland duct); PR (x400), p53 (x400).

**Figure 3:**
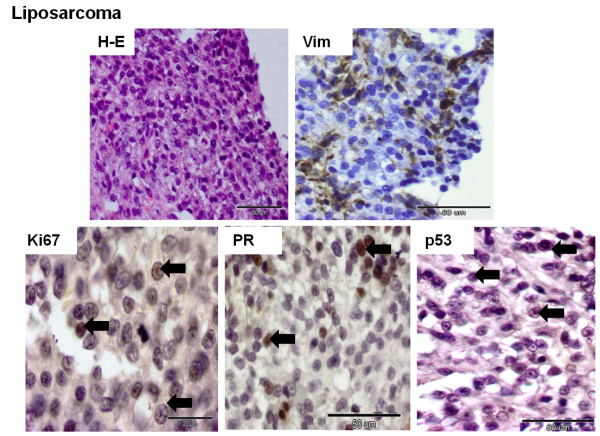
**Histological and immunohistochemical images of canine mammary liposarcoma.** The histological sample was stained with the standard hematoxylin and eosin (H-E) method. Immunopositivity (nuclear or cytoplasmic) is shown as brown precipitate in neoplastic cells. The EnVision + System-HRP detection system was used, and the signal was visualized with chromogen 3,3-diaminobenzidine 3-3' (DAB). Arrows indicate positive nuclear staining of cells. The images were obtained under the Olympus BX41 microscope. Original magnification: H-E (x400), Vim (x400), Ki67 (x1000), PR (x400), p53 (x400).

**Figure 4:**
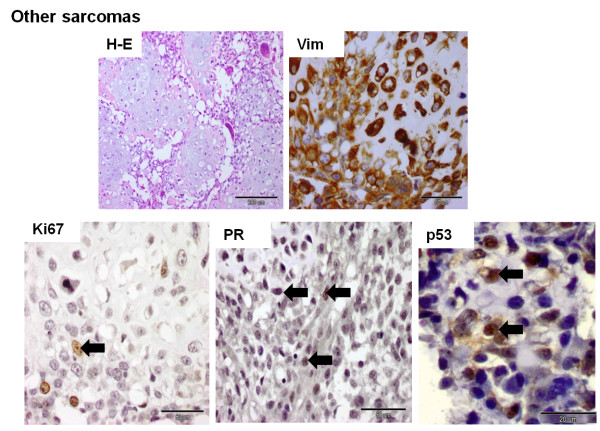
**Histological and immunohistochemical photographs of other canine mammary sarcomas.** The histological sample was stained with the standard hematoxylin and eosin (H-E) method. Immunopositivity (nuclear or cytoplasmic) is shown as brown precipitate in neoplastic cells. The EnVision + System-HRP detection system was used, and the signal was visualized with chromogen 3,3-diaminobenzidine 3-3' (DAB). Arrows indicate positive nuclear staining of cells. The images were obtained under the Olympus BX41 microscope. Original magnification: H-E (x100), Vim (x400), Ki67 (x400), PR (x400), p53 (x1000).

**Figure 5:**
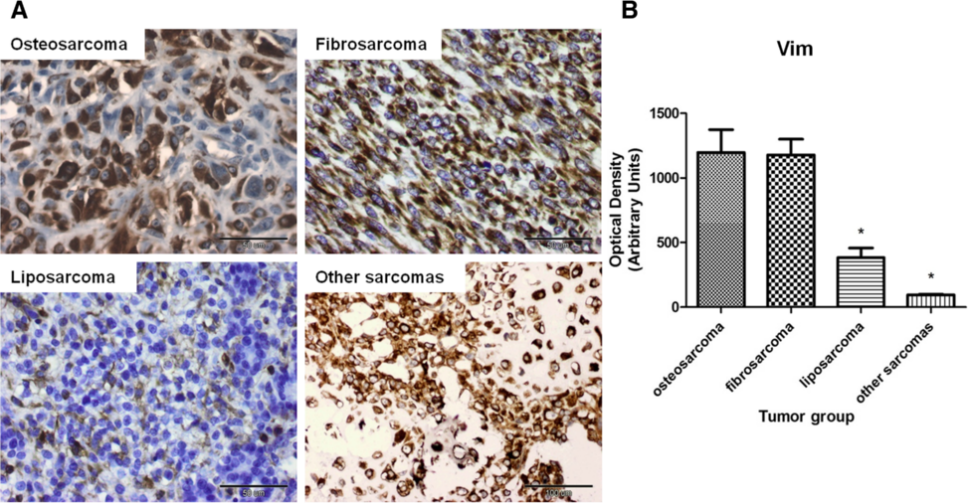
**Vimentin (Vim) expression in canine mammary sarcomas of various histological type. (A)** Representative images of CMSs obtained under the Olympus BX41 microscope. Positive staining for Vim is observed as brown precipitate in the cytoplasm of neoplastic cells. The EnVision + System-HRP detection system was used, and the signal was visualized with chromogen 3,3-diaminobenzidine 3-3' (DAB). **(B)** The graph represents integrated optical density (IOD) of vimentin-positive cells in canine mammary sarcomas. The colorimetric intensity of IHC-stained antigen spots was determined in a computer-assisted image analyzer (Olympus Microimage™ Image Analysis version 4.0 for Windows, USA), and the color intensity of the antigen spot is expressed as mean pixel optical density on a 1–256 scale. The results are presented as the mean (±SEM) from all tumors in each group. Data was processed in Prism 5.00 software (GraphPad Software, California, USA) using one-way ANOVA and Tukey's HSD post-hoc test. P-values <0.05 (*) were regarded as significant and marked with an asterisk (*).

**Figure 6:**
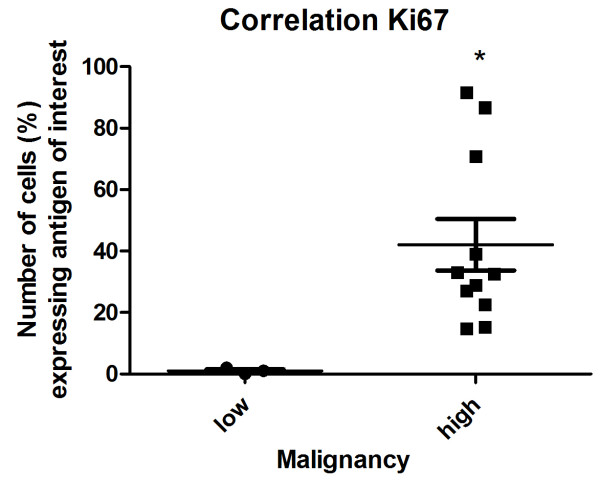
**Proliferation activity based on the mean values of the Ki67 index and its correlation with malignancy grade.** Data was processed by ANOVA + Tukey HSD post-hoc tests (Graph Pad Prism 5.0). P-values <0.05 (*) were regarded as significant and marked with an asterisk (*).

The staining pattern was evaluated in sarcomatous areas of the tumor (Figures 1, 2, 3 and 4). All 18 cases were positive for Vim. The mean optical density related to Vim expression in liposarcomas and other sarcomas was significantly lower than in osteosarcomas and fibrosarcomas (*p* <0.05) (Figure 5A, 5B). Focal moderate (+) expression of α-SMA was found in osteosarcomas (n = 3) and fibrosarcomas (n = 3) (Figures 1 and 2). Moderate cytoplasmic expression of α-SMA and Des was observed in one fibrosarcoma cell (Figure 2). The expression of CK and p63 was not observed in neoplastic cells. Ki67 was expressed by all CMSs, p53 – by 50% CMSs (n = 9), PR – by 27.8% (n = 5), and ERα expression was reported only in one sample (Figures 1, 2, 3 and 4). Detailed data is given in Tables 1 and 2. Spearman's analysis revealed that Ki67 expression (*p* = 0.034) was significantly correlated with the CMS grade (Figure 6). No significant correlation was found between proliferation markers (Ki67 expression and MI) and the expression levels of hormone receptors ERα and PR.

**Table 1:** Immunoreactivity for intermediate filaments and p63 in the analyzed CMS groups

**Immunoreactivity for intermediate filaments and p63**																					
**Group of CMS**	**N**	**Vim**				**CK**				**α-SMA**				**Des**				**p63**			
		**-**	**±**	**+**	**++**	**-**	**±**	**+**	**++**	**-**	**±**	**+**	**++**	**-**	**±**	**+**	**++**	**-**	**±**	**+**	**++**
Osteosarcoma	8	0	0	0	8	8	0	0	0	4	1	3	0	8	0	0	0	8	0	0	0
Fibrosarcoma	6	0	0	0	6	6	0	0	0	3	0	3	0	4	1	1	0	6	0	0	0
Liposarcoma	2	0	0	0	2	2	0	0	0	1	1	0	0	2	0	0	0	2	0	0	0
Other sarcomas	2	0	0	0	2	2	0	0	0	2	0	0	0	2	0	0	0	2	0	0	0

N = number of cases, CMS = canine mammary sarcoma, Vim = vimentin, CK = cytokeratin, α-SMA =  alpha smooth muscle actin, Des = desmin. The number of immunoreactive cells was classified as: - = none; ± = slight (positive cells constituted less than 10%); + = moderate (10-50% of cells were positive); and ++ = intense (more than 50% of cells were positive).

**Table 2:** Frequency of positively stained cases of CMS investigated for the expression of Ki67, ERα, PR and p53 according to histological type and grade

**Frequency of Ki67, ERα, PR and p53 immunopositivity**					
**Group of CMS**	**N**	**Ki67 N(%)**	**ERα N(%)**	**PR N(%)**	**p53 N(%)**
Osteosarcoma	8	8(100)	1(12.5)	1(12.5)	4(50)
Fibrosarcoma	6	6(100)	0(0)	2(33.3)	3(50)
Liposarcoma	2	2(100)	0(0)	1(50)	1(50)
Other sarcomas	2	2 (100)	0(0)	1(50)	1(50)
Grade of CMS	N				
High-grade	16	16(100)	1(6.3)	4(25)	9(56.3)
Low-grade	2	2(100)	0(0)	1(50)	1(50)
Total	18	18(100)	1 (5.6)	5(27.8)	9(50)

N = number of cases.

## Discussion

Canine mammary sarcomas (CMSs) are a rare type of tumors, and little is known about their biology. Our results provide new insights into clinical data regarding CMSs. Researchers are divided over the frequency of CMS occurrence. Previous research demonstrated that CMSs accounted for a small percent (0.45-16.7%) of CMTs [5,28,29], and similar results were reported in our study. According to some authors, CMSs constitute 40% of all mammary malignancies [29,30]. It should be noted, however, that in selected analyses, carcinosarcomas were included in the group of sarcomas [31]. In this study, CMSs were observed in older female dogs, which is consistent with reports describing the mean age at CMT diagnosis [3,32]. One study found that younger dogs (mean age of 9 years) were more likely to be affected by CMSs than canine mammary carcinomas [5], but other morphological features were similar to those reported by Misdorp et al. [5]. Interestingly, we noted that CMSs were more likely to affect the left rather than the right mammary gland. However, considering the small number of samples and the scarcity of published data, our results could be purely coincidental. In view of previous reports indicating that the location of the tumor had no effect on the outcome, the above information has no clinical significance [2,32].

Selected studies indicated that purebred dogs were more predisposed to mammary tumors than mongrels [33]. In a survey of 101 CMTs, one case of sarcoma was observed in small-breed dog and another in a different dog breed [34]. In our study, the majority of CMSs were diagnosed in large-breed dogs. To date, only one study has demonstrated that CMSs were common in medium- and large-breed dogs [11]. In our opinion, the fact that CMSs are most frequently observed in large-breed dogs could be attributed to the high popularity of those breeds in Poland. Due to a limited number of cases, breed predilection for only CMSs has not been established, but it has been reported for all canine mammary tumors [1,28,33].

In the retrospective analysis, fibrosarcoma was the most frequent type of sarcoma in the group of malignant CMTs. Our results corroborate the findings of other authors [5,35]. Gómez et al. [35] described fibrosarcoma as the second most common malignant CMT after carcinoma. Fibrosarcoma affected 1.3-5.56% of the populations surveyed in different studies [36,37].

The fact that sarcomas are malignant tumors is commonly accepted. Several studies described malignant progression from complex carcinomas to simple carcinomas and sarcomas [1,32]. In our study, the majority (88.9%) of the examined CMSs were high-grade tumors, but no significant correlation was observed between the histological type and the grade of CMSs. The above could be attributed to the small number of samples and the low statistical power to detect differences.

The origin of mesenchymal tumors and mesenchymal elements (in particular cartilage and bone) in the mammary gland has been under debate for many years. Some authors suggested that mesenchymal components originated from myoepithelial cells [20] or pluripotent stem cells [38]. Studies of CMT histogenesis examined the expression of microfilaments [1,6,9,17]. Analyses of intermediate filament expression support differentiation between canine mammary sarcomas (in particular fibrosarcomas and spindle cell carcinomas or malignant myoepitheliomas and hemangiopericytomas). All CMSs examined in our study showed expression of Vim (at various levels) regardless of their histological type, which could indicate that they originated from mesenchymal stem cells [14,39]. Various expression levels of Vim could be related to differences in CMS structure. Terra et al. [40] recently reported significantly higher expression of vimentin in malignant canine mammary tumors than in benign lesions. To the best of our knowledge, there are no published reports regarding CMSs. In this study, the expression of α-SMA was observed in three osteosarcomas and three fibrosarcomas, and Des expression was noted in one case of fibrosarcoma. Those observations could point to myofibroblastic focal differentiation that was previously described in feline mammary and breast sarcomas [41,42]. Some authors suggested the myoepithelial origin of myofibroblasts [43], but the absence of CK and p63 immunoreactivity combined with strong Vim expression in this study points to the mesenchymal nature of CMSs. Myofibroblastic differentiation was observed in breast malignancies (malignant fibrous histiocytoma, low-grade myofibroblastic sarcoma) [42,44], but it was not noted in mammary tumors in animals.

In this study, CK expression was not observed in any of the examined CMSs. Our findings are consistent with previously published reports where no direct transitions between carcinoma and sarcoma were noted [13,14]. In two reports [9,17], CK expression was observed in canine mammary osteosarcomas, and it was limited to epithelial-like cells.

In the present study, high levels of Ki67 expression were noted in the examined CMSs. In other studies, cell proliferation activity varied significantly across different histologic grades [15,45]. In our study, the expression of Ki67 was correlated with the sarcoma grade. According to many authors, proliferative activity can predict the biological behavior of canine mammary carcinomas: metastases, disease-free survival (DFS) and overall survival (OS) [15,21,45,46]. Canine mammary sarcomas were characterized by higher levels of Ki67 expression than carcinomas [21].

To the best of our knowledge, the expression of p53 in CMSs has been investigated by very few authors [47,48]. Similar expression levels of p53 in various types of CMSs (mostly high grade sarcomas) were demonstrated in this study. This is a relative new observation, and to date, p53 expression has been demonstrated mainly in osteosarcomas [47,49]. Our findings could suggest that p53 plays a role in malignant progression of the tumor [27,50]. Overexpression of p53 has been described in 50% of breast sarcomas [50]. Those results largely corroborate our findings. Interestingly, other authors have suggested the possible prognostic role of p53 expression in breast sarcomas [50]. Similar results were noted in this study, but the utility of p53 as a prognostic maker should be examined on a larger number of samples and in view of follow-up data.

Most of the examined CMSs showed no expression of ERα or PR. In literature, high levels of ERα and PR have been observed in well-differentiated tumors with a low proliferation rate [21,22]. Hormonal therapy is thought to be beneficial in those types of tumors [22,51]. As expected, although the majority of CMSs were hormone-independent, ERα and PR expression was not correlated with tumor type or grade. Those findings support our observations that hormonal treatment, which is often recommended in mammary carcinomas [24], could be ineffective in sarcomas due to an absence of hormone receptors [52]. The differences between the hormonal status of carcinomas and sarcomas could be attributed to variations in their histogenetic origin. Our study demonstrated an absence of correlations between ERα and PR expression and proliferation status. Hormone receptors did not show antiproliferative activity that is observed in mammary cancers [46,51].

## Conclusions

According to our best knowledge, this paper makes the first attempt to comprehensively analyze CMSs. Our study demonstrated that immunohistochemical analyses support differential diagnosis of those rare tumors. Higher expression levels of Vim were correlated with the histological type of the evaluated sarcoma. Ki67 expression was associated with tumor grade. Further work is required to validate our results on a larger number of samples.

## Competing interests

The authors declare no financial or non-financial competing interests.

## Authors’ contributions

ID: research design, collection of research material, histopathological and immunohistochemical examination of canine mammary gland tumors, assessment of immunohistochemical results, manuscript preparation, figure preparation; RS: histopathological examination of canine mammary gland tumors, assessment of immunohistochemical results; MK: statistical analysis, manuscript revision. The authors have read and approved the final manuscript.
